# Perinatal Outcomes and Risk Factors for Preterm Birth in Twin Pregnancies in a Chinese Population: A Multi-center Retrospective Study

**DOI:** 10.3389/fmed.2021.657862

**Published:** 2021-04-21

**Authors:** Sijian Li, Jinsong Gao, Juntao Liu, Jing Hu, Xiaoxu Chen, Jing He, Yabing Tang, Xinghui Liu, Yinli Cao

**Affiliations:** ^1^Department of Obstetrics and Gynecology, Peking Union Medical College, Peking Union Medical College Hospital, Chinese Academy of Medical Sciences, National Clinical Research Center for Obstetric & Gynecologic Diseases, Beijing, China; ^2^Department of Obstetrics and Gynecology, School of Medicine, Women's Hospital, Zhejiang University, Zhejiang, China; ^3^Department of Obstetrics and Gynecology, Hunan Maternal and Child Health Care Hospital, Changsha, China; ^4^Department of Obstetrics and Gynecology, Sichuan University West China Second Hospital, Chengdu, China; ^5^Department of Obstetrics and Gynecology, Northwest Women and Children's Hospital, Xi'an, China

**Keywords:** perinatal outcomes, preterm birth, risk factors, twin pregnancies, Chinese population

## Abstract

**Background:** Twin pregnancies are associated with an increased risk of adverse maternal and neonatal outcomes, mainly owing to prematurity. Few studies have evaluated the risk factors for preterm birth (PTB) in Chinese population. The objective of this study is to present the short-term maternal-neonatal outcomes, investigating the potential risk factors associated with preterm birth in Chinese twin pregnancies.

**Methods:** A multi-center retrospective study of women pregnant with twins ≥28 weeks of gestation was conducted. Maternal and neonatal outcomes were analyzed. Logistic regression was used to identify potential risk factors for PTB before 37, 34, and 32 weeks, respectively.

**Results:** A total of 3,288 twin pregnancies and 6,576 neonates were included in 99,585 pregnancies. The rate of twin pregnancy was 3.3%, while the PTB rate before 37, 34, and 32 weeks among this population were 62.1, 18.8, and 10.4%, respectively. Logistic regression revealed that monochorionicity [Odds ratio (OR) 3.028, 95% confident interval (CI) 2.489–3.683, *P* < 0.001], gestational weight gain (GWG) <10 kg (OR 2.285, 95% CI 1.563–3.339, *P* < 0.001) and GWG between 10 and 15 kg (OR 1.478, 95% CI 1.188–1.839, *P* < 0.001), preeclampsia (PE) (OR 3.067, 95% CI 2.142–4.390, *P* < 0.001), and intrahepatic cholestasis of pregnancy (ICP) (OR 3.122, 95% CI 2.121–4.596, *P* < 0.001) were the risk factors for PTB before 37 weeks. Monochorionicity (OR 2.865, 95% CI 2.344–3.501, *P* < 0.001), age < 25 years (OR 1.888, 95% CI 1.307–2.728, *P* = 0.001), and GWG <10 kg (OR 3.100, 95% CI 2.198–4.372, *P* < 0.001) were risk factors for PTB before 34 weeks. Monochorionicity (OR 2.566, 95% CI 1.991–3.307, *P* < 0.001), age younger than 25 years (OR 1.964, 95% CI 1.265–3.048, *P* = 0.003), and GWG <10 kg (OR 4.319, 95% CI 2.931–6.364, *P* < 0.001) were the risk factors for PTB before 32 weeks.

**Conclusions:** Monochorionicity and GWG <10 kg were two major risk factors for PTB before 32, 34, and 37 weeks, whereas maternal age, PE, and ICP were also risk factors for PTB in specific gestational age.

## Introduction

Globally, twin pregnancies account for ~2–4% of all births and it has been reported that the incidence has increased dramatically over the past few decades (1–4). Patients with twin gestation are more likely to suffer pregnancy complications, such as anemia, hypertensive disorders, gestational diabetes mellitus, and postpartum hemorrhage, than those with singleton gestation (5, 6). Compared with the singleton gestation, twin gestation is associated with a significantly increased risk of perinatal maternal and fetal morbidity and mortality (7–9). The risk of potentially life-threatening conditions, maternal near-miss, severe maternal outcomes, and maternal deaths among twin pregnancies were 2 to 4-fold higher than those for singleton gestation (7). Prematurity and its related complications are the leading cause of mortality in children <5 years of age (10). Previously studies demonstrated for twin gestations, the rates of preterm birth at <37 and <32 weeks are 5.7 and 7.1 times higher, respectively, than those for singleton gestations: with the rate of preterm birth in twin gestation ranging from 31 to 63% (8, 9, 11). The incidence of preterm birth in twin gestation has also showed an increasing trend over the past decades as the rate of twin pregnancies accelerates, thereby leading to more public health challenges (3).

A better understanding of perinatal maternal and neonatal complications and outcomes of twin pregnancies will undoubtedly enhance clinical practices. In addition, the proper identification of patients at higher risk of preterm birth would enable optimization of the available interventions to reduce the adverse perinatal outcomes associated with preterm birth. Meanwhile, the availability of a risk stratification system will help to decrease unnecessary interventions in low-risk patients and improve the therapeutic efficiency in those at high risk of preterm birth. Methods for predicting preterm birth and risk factors for preterm birth in twin pregnancy have long been studied. A history of preterm birth (12), maternal clinical characteristics, such as age and height (13, 14), gestational weight gain (GWG) (15, 16), maternal complications, such as preeclampsia (17), biophysical and biochemical tests, (18) are all proposed to aid in identifying and optimizing management of preterm birth in twin gestation.

However, research involving twin pregnancies is mainly conducted in western countries, and data on the perinatal outcomes of twin pregnancies among Chinese women are limited; few studies have evaluated the risk factors for preterm birth in this large population. We aimed to analyze the maternal and neonatal outcomes among Chinese twin pregnancies in a large multi-center cohort and to identify the potential risk factors for preterm birth in this population.

## Materials and Methods

### Data Sources, Inclusion and Exclusion Criteria, and Definitions

This study was conducted in 14 representative medical centers (including two secondary and 12 tertiary, seven general hospitals, and seven maternal and child health care centers) from 10 provinces in the four major economic regions of China from October 1, 2016 to September 30, 2017. Complete medical information for each birth, including maternal demographics, medical and obstetric history, and maternal and perinatal outcome information were retrospectively registered into a prospectively designed network-based standardized data acquisition system obtained from digital and written medical records. The medical records from the 14 participating hospitals in this study were standardized before the research was initiated. This retrospective birth registry study was approved by the Ethics Committee of Peking Union Medical College Hospital (approval number: PUMCH-JS-1151). The need for informed consent was waived due to the retrospective nature of the study. All pregnant women aged 18–55 years, who had their first antenatal visit in the first trimester and gave birth to twins ≥28 weeks of gestation, were eligible for inclusion. Women without data on the exact age and gestational age at delivery were excluded. Data including demographic factors, clinical characteristics including maternal height and weight, GWG, methods of conception, chorionicity, gestational age at delivery, pre-pregnancy and pregnancy complications, medical treatment, pregnancy outcomes, and mode of delivery were collected from the medical records and confirmed by the treating physician. Neonatal birth weight, length, Apgar scores at 1, 5, and 10 min, outcomes (transfer to neonatal intensive care unit, discharge from hospital, or neonatal death within 7 days after delivery) were recorded.

A total of 3,288 patients with twin pregnancies and 6,576 neonates met the inclusion criteria among the 99,585 pregnant women who were enrolled. In this study, preterm birth is defined as birth before 37 completed weeks of gestation according to the World Health Organization (WHO) (19). Early preterm birth and very early preterm birth are defined as birth before 34 and 32 completed weeks of gestation, respectively. Gestational age (GA) among the patients included in our study was calculated from the date of the last menstrual period (LMP) and adjusted using ultrasound measurements. Ultrasound examination was performed when the patients were confirmed as pregnant for the first time (at 6–9 weeks according to the LMP). The second ultrasound examination was performed at 11–13 6/7 weeks of gestation. If patients missed these two examinations to calculate the estimated due date, an additional ultrasound examination was administrated at the beginning of the second trimester (usually at 20 weeks of gestation, not exceeding 21 6/7 weeks) to adjust the GA. If pregnancy resulted from assisted reproductive technology (ART), the ART-derived GA was used to assign the estimated due date. The adjusted methods for estimating the due date were in accordance with the guideline (20). The chorionicity was defined using ultrasonography during pregnancy according to previous researches (21). Those with uncertain chorionicity in the first trimester repeated the ultrasonography in the second or third trimester. The chorionicity of all such patients were reexamined after delivery in this study. GWG was calculated by subtracting the self-reported pre-pregnancy weight, or if unavailable, the first-measured weight at the antenatal visit, from the last-measured weight closest to delivery, similar to a previous study's description (22). GWG was divided into categories including GWG <10 kg, GWG between 10 and 14.99 kg, GWG between 15 and 19.99 kg, and GWG more than 20 kg. Pre-pregnancy body mass index (BMI) was categorized as underweight (BMI <18.5 kg/m^2^), normal (18.5–23.99 kg/m^2^), overweight (24–27.99 kg/m^2^), and obese (BMI ≥ 28 kg/m^2^) according to the Chinese BMI categorization (23).

Pre-pregnancy complications referred to a history of preterm birth or late abortion or other related comorbidities such as thyroid disorders, diabetes mellitus, hypertension, liver diseases, iron deficiency anemia, autoimmune diseases, and heart disease. Pregnancy complications included gestational hypertension, preeclampsia superimposed on chronic hypertension, preeclampsia, and eclampsia, gestational diabetes mellitus (GDM), and intrahepatic cholestasis of pregnancy (ICP), and premature rupture of membrane (PROM). Hypertensive disorders of pregnancy (HDP) were classified according to the ISSHP classifications (24). Delivery complications included postpartum hemorrhage, amniotic fluid embolism. Postpartum hemorrhage was defined as cumulative blood loss ≥500 mL in vaginal delivery or ≥1,000 mL in cesarean section within 24 h after delivery (25). Neonatal low birth weight (LBW) was defined as neonatal birth weight <2,500 g, whereas birth weight <1,500 and <1,000 g was classified as very low birth weight (VLBW) and extremely low birth weight (ELBW), respectively. Small for gestational age (SGA) was defined as an infant weighing less than the 10th centile birth weight for gestational age and sex. Neonatal asphyxia referred to Apgar score <7 at 1 min or Apgar score <7 at 5 min after delivery. The selective intrauterine growth restriction (sIUGR) was defined as an estimated fetal weight of one fetus below the 10th centile and an intertwin birthweight discordance of ≥ 25% (26). Twin to twin transfusion syndrome (TTTS) was defined as a deepest vertical pocket (DVP) of amniotic fluid > 8 cm in the amniotic sac of the recipient twin and a DVP <2 cm in the donor twin (27). Fetal respiratory distress syndrome (RDS) was diagnosed by the presence of clinical signs such as grunting, flaring, tachypnea, retractions, and requiring respiratory support (supplemental oxygen requirement or non-invasive/invasive ventilation) and admission to a neonatal intensive care unit (NICU) for respiratory support.

### Outcomes Measurement, Timing of Planned Delivery, and Follow-Up During Pregnancy in Our Study

The main outcome of the study was the preterm birth before 37, 34, and 32 weeks in twin pregnancy. The secondary outcomes included maternal pregnancy and delivery complications, and neonatal outcomes. The neonatal outcomes included LBW, SGA, neonatal asphyxia, sIUGR, RDS, admission to NICU, pneumonia, neonatal infection, retinopathy of prematurity (ROP), necrotizing enterocolitis (NEC), and major congenital malformations.

The schedule used for planned delivery in our study was according to the Chinese guideline on the management of twin pregnancy. Uncomplicated monochorionic diamniotic twin gestations underwent delivery between 34 and 37 weeks of gestation and uncomplicated monochorionic monoamniotic twin gestations underwent delivery between 32 and 34 weeks of gestation. In those patients with uncomplicated dichorionic diamniotic twin gestations, we terminated the pregnancy after 37 weeks of gestation. In cases of patients with other complications such as HDP or ICP, the timing of the planned delivery was adjusted according to those comorbidities. Women with mild preeclampsia or chronic hypertension, gestational hypertension were planned delivery after 37 weeks of gestation. The pregnancy was terminated if the condition was unstable or reached 34 weeks of gestation in patients with severe preeclampsia. At ≥34 weeks of gestation, pregnancy was terminated if there were serious complications such as placental abruption, and intrauterine fetal death that threatened maternal and neonatal safety. Pregnancy was also terminated at ≥34 weeks if fetal growth was restricted and accompanied by abnormal umbilical cord blood flow and oligohydramnios. In mild ICP, pregnancy was terminated between 38 and 39 weeks of gestation. In severe ICP, the pregnancy was terminated between 34 and 37 weeks of gestation, but the treatment response and presence of a combination of fetal distress, twin births, or other maternal complications were taken into account.

All participants were regularly followed-up by the perinatal health care management teams in corresponding hospitals. The framework for maternal antenatal care was consistent with WHO recommendations and Chinese practical guidelines (28). Patients with advanced maternal age or complications received more closed follow-up till delivery. Additional antenatal care was given in case of emergency situations such as intrauterine fetal distress, decreased fetal movements, or elevated blood pressure.

### Statistical Analysis

Continuous variables are described by means ± standard deviation (range) if they were normally distributed or as medians and interquartile ranges (IQRs) if they were abnormally distributed. Discrete variables are expressed as counts (percentage). Variables were compared between the two outcomes (preterm birth or full-term birth) using univariate analysis. Continuous variables were compared using the Student's *t*-test or the Mann-Whitney U-test, depending on their distribution. Categorical variables were compared using the chi-squared test or Fisher's exact test. Variables in the univariate analysis with *P*-values < 0.2 were selected for potential inclusion in the multivariate logistic regression. Forward, stepwise logistic regression was performed to determine potential risk factors for preterm birth. Odds ratios (OR) with 95% confidence intervals (CI) and *P*-values were calculated. A two-tailed *P*-value < 0.05 was considered significant. Statistical analysis was conducted using SPSS (Version 21.0; SPSS Inc., Chicago, IL, USA) or GraphPad Prism (Version 8.0) software.

## Results

### Maternal Clinical Characteristics and Complications During Pregnancies

The rate of twin pregnancy was 3.3% in our cohort (mean age: 30.9 ± 4.4 years (range: 18–53 years), mean height: 161.3 ± 4.8 cm). Natural conception occurred in 60.3% of participants, whereas the remaining 39.7% conceived using ART. Sixty-eight patients had a history of preterm birth, with 23 experiencing preterm birth two times or more. Dichorionic diamnioticity (DCDA, 74.1%) was the most predominant twin subtype, followed by monochorionic diamnioticity (MCDA, 25.6%), and monochorionic monoamnioticity (MCMA, 0.3%). The rate of preterm birth < 37, 34, and 32 weeks among this population was 62.1, 18.8, and 10.4%, respectively, with an average gestation of 35.6 ± 2.4 weeks. The incidence of spontaneous and iatrogenic preterm birth before 34 and 32 weeks was 5.0 and 2.8%, and 13.8 and 7.6%, respectively. Other maternal characteristics are listed in Table 1 and Supplementary Table 1.

**Table 1:** Maternal clinical characteristics and outcomes.

		***N***		***N***	**Percentile**
Age (y)	30.9 ± 4.4 (18–53)	3,288	DM in pregnancy	532	16.2%
Parity		3,288	GDM	518	
Nulli	2,122 (64.5%)		Type I DM	7	
Pluri	1,166 (35.5%)		Type II DM	7	
Height	161.3 ± 4.8 cm	3,148	Hyperthyroidism	30	0.9%
Method of Conception		3,288	Hypothyroidism	197	6.0%
Nature	1,982 (60.3%)		HDP	416	12.7%
ART	1,306 (39.7%)		No	2,872	
Chorionicity		3,288	Gestational hypertension	105	
DCDA	2,434 (74.1%)		cHTN	7	
MCDA	843 (25.6%)		PE superimoposed on cHTN	13	
MCMA	11 (0.3%)		PE	291	
Prepregnancy Weight	55.8 ± 9.0 kg	2,982	ICP	206	6.3%
Prepregnancy BMI	21.4 ± 3.2 kg/m^2^		PPROM	342	10.4%
Delivery weight	72.8 ± 10.9 kg	3,007	PPH	109	3.3%
Weight Gain	17.5 ± 5.6 kg		VD ≥ 500 ml	18	
Preterm birth history		68	VD ≥ 1,000 ml	8	
Iron diffciency anemia		233	CS ≥ 1,000 ml	83	
Gestational age	35.6 ± 2.4weeks	3,288	Management of PPH		
<32 weeks	341 (10.4%)		AIE	7	
32–33^+6^ weeks	276 (8.4%)		IIAE	2	
34–36^+6^ weeks	1,426 (43.4%)		UAE	5	
≥37 weeks	1,245 (37.9%)		Uterine B-lynch suture	43	
Mode of delivery		3,288	Uterine gauze packing	35	
VD	294 (8.9%)		Balloon tamponade packing	20	
CS	2,994 (91.1%)		Uterine arterial ligation	25	
Emergency CS	491 (16.4%)		Hysterectomy	2	

*Some variables were unavailable in some patients*.

*ART, assistant reproductive technology; DCDA, dichorionic diamniotic; MCDA, monochorionic diamniotic; MCMA, monochorionic monoamniotic; BMI, body mass index; VD, vaginal delivery; CS, cesarean section; DM, diabetes mellitus; GDM, gestational diabetes mellitus; HDP, hypertensive disorders of pregnancy; cHTN, chronic hypertension; PE, preeclampsia; ICP, intrahepatic cholestasis of pregnancy; PPROM, premature rupture of membrane; PPH, postpartum hemorrhage; AIE, arterial intervention embolism; IIAE, internal iliac arterial embolism; UAE, uterine arterial embolism*.

Pre-pregnancy complications and complications during pregnancy are also shown in Table 1 and Supplementary Table 1. Hypertension, diabetes mellitus, heart and liver diseases, autoimmune disease, hyperthyroidism, hypothyroidism, and iron deficiency anemia were documented. Diabetes mellitus (DM: 532, 16.1%), including GDM (518, 15.7%) and DM diagnosed before conception (14, 0.4%), were the most common complications. PROM occurred in 404 patients (term PROM, 62; preterm PROM, 342) before 37 weeks. Other common complications included HDP (416, 12.7%), iron deficiency anemia (233, 7.1%), hypothyroidism (197, 6.0%), and ICP (206, 6.3%). Hyperthyroidism (30, 0.9%), autoimmune diseases (9, 0.3%), arrhythmia (6, 0.2%), and acute fatty liver of pregnancy (AFLP, 4, 0.1%) were less common in our study. Viral hepatitis was also recorded (hepatitis B: 86, hepatitis C: 3). There were 96 cases of placenta previa, 193 cases of placenta accreta, and 46 patients who had both complications. In addition, placental abruption and retained placenta occurred in 27 and two patients, respectively. Umbilical cord abnormality occurred in 399 cases (cord around neck: 391, prolapse of cord: 8).

Delivery related complications were also recorded. One hundred and nine patients experienced postpartum hemorrhage (PPH, 3.3%), and 91 were severe cases defined as blood loss of >1,000 mL within 24 h after delivery. Management for PPH included arterial intervention embolism (7 cases), uterine B-lynch suture (43 cases), uterine packing (55 cases), uterine arterial ligation (25 cases), and a total hysterectomy (2 cases). Some patients received more than one method of treatment for PPH in our study. There were twin-related complications in 130 patients (57 were complicated with sIUGR, 6.8% of the MCDA pregnancies; the stillbirth of one fetus: 33, TTTS: 30). Three of the cases of the stillbirth of one fetus were complicated by TTTS. One case of fetal to maternal transfusion syndrome was also noted. No amniotic fluid embolism occurred.

After delivery, 12 women were transferred to the intensive care unit but there was no fatality.

### Neonatal Outcomes

The mean weight of the 6,576 neonates enrolled in the study was 2,369.3 ± 523.4 g, and their average length was 45.8 ± 3.6 cm. The median birth weight and length were 2,426 g and 47 cm, respectively. The 5, 10, 25, 75, 90, and 95 percentiles were 1,370, 1,650, 2,090, 2,702, 2,950, and 3,100 g for birth weight and 39, 40, 45, 48, 50, and 50 cm, for birth length. The incidence of SGA was 3.7% (204) in our research. Two hundred and eighty-four (4.3%) newborns were diagnosed with asphyxia on delivery, and the incidence of RDS was 1.1% (75 cases). The incidence of Apgar scores <7 and 5 at 1 min was 4.3 and 1.5%, respectively. The average Apgar scores at 1, 5, and 10 min were 9.25 ± 0.8, 9.51 ± 0.9, and 9.60 ± 1.2, respectively. The rate of normal birth weight, low birth weight, very low birth weight, and extremely low birth weight were 44.8 48.3, 6.1, and 0.9%, respectively. Almost half of the newborns were transferred to the neonatal intensive care unit (NICU, 43.81%) and 55.5% were uneventfully discharged. Pneumonia (5.0%) and neonatal infection (3.3%) were two common complications among neonates. Respiratory distress syndrome (1.1%), congenital deformity (0.6%), retinopathy of prematurity (0.8%), and necrotizing enterocolitis (2 cases) were also recorded. The perinatal death rate was 0.7% (Clinical characteristics and short-term outcomes were listed in Table 2).

**Table 2:** Short-term neonatal outcomes.

	**Male (*n* = 3,377)**	**Female (*n* = 3,199)**	**Total (*n* = 6,576)**
Weight (g)	2,401.4 ± 533.5	2,335.3 ± 510.5	2,369.3 ± 523.4
Length (cm)	45.9 ± 3.7	45.7 ± 3.6	45.8 ± 3.6
**Apgar scores**			
Apgar 1 min	9.25 ± 1.2	9.26 ± 1.1	9.25 ± 0.8
Apgar 5 min	9.48 ± 1.0	9.54 ± 0.86	9.51 ± 0.9
Apgar 10 min	9.61 ± 0.82	9.60 ± 0.85	9.60 ± 1.2
**Outcomes**			
NICU	1,424 (42.4%)	1,457 (45.5%)	2,881 (43.8%)
Discharge	11,925 (57.0%)	1,923 (53.9%)	3,648 (55.5%)
Death	28 (0.8%)	19 (0.6%)	47 (0.7%)
**LBW**			
No	1,620 (48.0%)	1,323 (41.4%)	2,943 (44.8%)
LBW	1,520 (45.0%)	1,655 (51.7%)	3,175 (48.3%)
VLBW	211 (6.2%)	188 (5.9%)	399 (6.1%)
ELBW	26 (0.8%)	33 (1.0%)	59 (0.9%)
SGA	117	125	204 (3.7%)
Asphyxia	145	139	284 (4.3%)
RDS	47	28	75 (1.1%)
Pneumonia	167	159	326 (5.0%)
Mechanical Ventilation	55	47	102 (1.6%)
Neonatal infection	101	115	216 (3.3%)
Congenital deformity	21	19	40 (0.6%)
CHD	19	15	34
ASD	8	7	15
VSD	6	5	11
PDA	3	2	5
Others	2	1	3
Polydactylism	1	2	3
Cleft	1	2	3
ROP	26	28	54 (0.8%)
NEC	1	1	2

*NICU, neonatal intensive care unit; LBW, low birth weight; VLBW, very low birth weight; ELBW, extremely low birth weight; SGA, small for gestation age; RDS, respiratory distress syndrome; CHD, congenital heart disease; ASD, atrial septal defect; VSD, ventricular septal defect; PDA, patent ductus arteriosus; ROP, Retinopathy of Prematurity; NEC, necrotizing enterocolitis*.

### Risk Factors for Preterm Birth in Twin Pregnancies

The results of the univariate and multivariate analyses identified risk factors associated with preterm birth before 37 weeks (Table 3), univariate analysis revealed that mochorionicity, maternal height, GWG, PE, and ICP were associated with preterm birth. Monochorionicity [Odds ratio (OR) 3.028, 95% confident interval (CI) 2.489–3.683, *P* < 0.001], GWG <10 kg (OR 2.285, 95% CI 1.563–3.339, *P* < 0.001) and GWG between 10 and 15 kg (OR 1.478, 95% CI 1.188–1.839, *P* < 0.001), PE (OR 3.067, 95% CI 2.142–4.390, *P* < 0.001), and (OR 3.122, 95% CI 2.121–4.596, *P* < 0.001) were the risk factors for preterm birth before 37 weeks in further multivariate logistic regression.

**Table 3:** Logistics regression to identify potential risk factors for preterm birth before 37 weeks (*N* = 3,288).

**Variables**	**PTB before 37weeks (*n* = 2,043)**	**Term after 37 weeks (*n* = 1,245)**	***P*-value**	**Multivariate analysis**		
				**OR**	**95% CI**	***P*-value**
Age (y)^*^			0.050			
<25	129 (6.3%)	57 (4.6%)				
25–34	1,484 (72.6%)	944 (75.8%)				
≥35	430 (21.0%)	244 (19.6%)				
Parity^*^			0.189			
Nulli	1,301 (63.7%)	821(65.9%)				
Pluri	742 (36.3%)	424(34.1%)				
PTB histroy			0.529			
Yes	45 (2.2%)	23 (1.8%)				
No	1,998 (97.8%)	1,222 (98.2%)				
Conception^*^			0.186			
Natural	1,250 (61.2%)	732 (58.8%)				
ART	793 (38.8%)	513 (41.2%)				
Chorionicity^*^			<0.001			
Dichorionic	1,359 (66.5%)	1,075 (86.3%)			Ref.	
Monochorionic	684 (33.5%)	170 (13.7%)		3.028	2.489–3.683	<0.001
Height (cm)^*^			<0.001			0.014
<160	649 (33.4%)	323 (26.8%)		1.171	0.985–1.392	0.074
160–169	1,238 (63.7%)	825 (68.5%)			Ref.	
≥170	56 (2.9%)	57 (4.7%)		0.642	0.424–0.973	0.037
Pre BMI (kg/m^2^)			0.887			
<18.5	298 (16.3%)	191 (16.5%)				
18.5–23.9	1,209 (66.2%)	761 (65.8%)				
24.0–27.9	256 (14.0%)	158 (13.7%)				
≥28.0	63 (3.5%)	46 (4.0%)				
Weight Gain (kg)^*^			<0.001			<0.001
<10	145 (7.8%)	44 (3.8%)		2.285	1.563–3.339	<0.001
10–14.9	412 (22.3%)	184 (15.9%)		1.478	1.188–1.839	<0.001
15–19.9	675 (36.5%)	470 (40.6%)			Ref.	
≥20	617 (33.4%)	460 (39.7%)		0.899	0.753–1.072	0.236
HDP^*^			<0.001			<0.001
No	1,740 (85.2%)	1,132 (90.9%)			Ref.	
HTN	4 (0.2%)	3 (0.2%)		0.704	0.151–3.271	0.654
GH	60 (2.9%)	45 (3.6%)		0.689	0.440–1.080	0.104
PE	239 (11.7%)	65 (5.2%)		3.067	2.142–4.390	<0.001
ICP^*^			<0.001			
Yes	172 (8.4%)	34 (2.7%)		3.122	2.121–4.596	<0.001
No	1,871 (91.6%)	1,211 (97.3%)			Ref.	

* *Factors assigned to multivariate logistic regression analysis*.

*Ref., reference; PTB, preterm birth; ART, assistant reproductive technology; BMI, body mass index; HDP, hypertensive disorders of pregnancy; HTN, hypertension; GH, gestational hypertension; PE, preeclampsia; ICP, intrahepatic cholestasis of pregnancy*.

The potential factors for predicting preterm birth before 34 weeks are shown in Table 4. Maternal age and height, methods of conception, chorionicity, GWG, HDP, and ICP were included in the multivariable logistic regression, Monochorionicity (OR 2.865, 95% CI 2.344–3.501, *P* < 0.001), age <25 years (OR 1.888, 95% CI 1.307–2.728, *P* < 0.001), and GWG <10 kg (OR 3.100, 95% CI 2.198–4.372, *P* < 0.001) remained statistically significant. Subgroup analysis concerning the impact of chorionicity on spontaneous and iatrogenic preterm birth before 34 weeks are shown in Supplementary Table 2. Monochorionicity was associated with increased risk of both spontaneous and iatrogenic preterm birth before 34 weeks, and the adjusted OR was 2.390 (95% CI 1.636–3.491, *P* < 0.001) and 3.037 (95% CI 2.427–3.800, *P* < 0.001), respectively.

**Table 4:** Logistics regression to identify potential risk factors for preterm birth before 34 weeks (*N* = 3,288).

**Variables**	**PTB before 34 weeks (*n* = 617)**	**after 34 weeks (*n* = 2,671)**	***P*-value**	**Multivariate analysis**		
				**OR**	**95% CI**	***P*-value**
Age (y)^*^			0.004			0.003
<25	52 (8.4%)	134 (5.0%)		1.888	1.307–2.728	0.001
25–34	439 (71.2%)	1,989 (74.5%)			Ref.	
≥35	126 (20.4%)	548 (20.5%)		1.107	0.870–1.409	0.408
Parity			0.513			
Nulli	391 (63.4%)	1,731 (64.8%)				
Pluri	226 (36.6%)	940 (35.2%)				
PTB histroy			1.000			
Yes	13 (2.1%)	55 (2.1%)				
No	604 (97.9%)	2,616 (97.9%)				
Conception^*^			0.171			
Natural	387 (62.7%)	1,595 (59.7%)				
ART	230 (37.3%)	1,076 (40.3%)				
Chorionicity^*^			<0.001			
Dichorionic	337 (54.6%)	2,097 (78.5%)			Ref.	
Monochorionic	280 (45.4%)	574 (21.5%)		2.865	2.344–3.501	<0.001
Height (cm)^*^			0.002			0.054
<160	204 (35.4%)	768 (29.9%)		1.069	0.868–1.316	0.532
160–169	362 (62.8%)	1,701 (66.1%)			Ref.	
≥170	10 (1.7%)	103 (4.0%)		0.400	0.181–0.883	0.023
Pre BMI (kg/m^2^)			0.345			
<18.5	92 (17.3%)	397 (16.2%)				
18.5–23.9	336 (63.3%)	1,634 (66.7%)				
24.0–27.9	85 (16.0%)	329 (13.4%)				
≥28.0	18 (3.4%)	91 (3.7%)				
Weight Gain (kg)^*^			<0.001			<0.001
<10	74 (13.7%)	115 (4.7%)		3.100	2.198–4.372	<0.001
10–14.9	127 (23.6%)	469 (19.0%)		1.215	0.939–1.571	0.138
15–19	199 (36.9%)	946 (38.3%)			Ref.	
≥20	139 (25.8%)	938 (38.0%)		0.667	0.524–0.849	0.001
HDP^*^			0.171			
No	528 (85.6%)	2,344 (87.8%)				
GH	17 (2.8%)	88 (3.3%)				
HTN	2 (0.3%)	5 (0.2%)				
PE	70 (11.3%)	234 (8.8%)				
ICP^*^			0.117			
Yes	30 (4.9%)	176 (6.6%)				
No	587 (95.1%)	2,495 (93.4%)				

* *Factors assigned to multivariate logistic regression analysis*.

*Ref., reference; PTB, preterm birth; ART, assistant reproductive technology; BMI, body mass index; HDP, hypertensive disorders of pregnancy; HTN, hypertension; GH, gestational hypertension; PE, preeclampsia; ICP, intrahepatic cholestasis of pregnancy*.

When we analyzed preterm birth <32 gestational weeks, factors that may affect preterm birth are summarized in Table 5. Univariate analysis showed that maternal age (*P* = 0.007), height (*P* = 0.013), chorionicity (*P* < 0.001), GWG (*P* < 0.001), and HDP (*P* = 0.034) were significantly associated with preterm birth before 32 weeks, whereas parity, HDP, and ICP were likely to predict preterm birth. These factors were further used for multivariable analysis, where monochorionicity (OR 2.566, 95% CI 1.991–3.307, *P* < 0.001), age younger than 25 years (OR 1.964, 95% CI 1.265–3.048, *P* = 0.003), and GWG <10 kg (OR 4.319, 95% CI 2.931–6.364, *P* < 0.001) were the risk factors for preterm birth before 32 weeks were statistically associated with increased risk for preterm birth before 32 weeks. Moreover, GWG between 10 and 15 kg slightly increased the risk of preterm birth before 32 weeks (OR 1.476, 95% CI 1.073–2.030, *P* = 0.017). The impact of chorionicity on spontaneous and iatrogenic preterm birth before 32 weeks was also conducted using multivariate logistic regression (Supplementary Table 3). Compared with dichorionicity, monochorionicity was associated with a higher risk of spontaneous preterm birth (OR 2.932, 95% CI 1.754–4.900, *P* < 0.001) and iatrogenic preterm birth (OR 2.510, 95% CI 1.872–3.367, *P* < 0.001).

**Table 5:** Logistics regression to identify potential risk factors for preterm birth before 32 weeks (total *N* = 3,288).

**Variables**	**PTB before 32 weeks (*n* = 341)**	**After 32 weeks (*n* = 2,947)**	***P*-value**	**Multivariate analysis**		
				**OR**	**95% CI**	***P*-value**
Age (y)^*^			0.007			0.010
<25	32 (9.4%)	154 (5.2%)		1.964	1.265–3.048	0.003
25–34	244 (71.6%)	2,184 (74.1%)			Ref.	
≥35	65 (19.1%)	609 (20.7%)		1.006	0.735–1.377	0.970
Parity^*^			0.073			
Nulli	205 (60.1%)	1,917 (65.0%)				
Pluri	136 (39.9%)	1,030 (35.0%)				
PTB histroy			0.841			
Yes	8 (2.3%)	60 (2.0%)				
No	333 (97.7%)	2,887 (98.0%)				
Conception			0.350			
Natural	214 (62.8%)	1,768 (60.0%)				
ART	127 (37.2%)	1,179 (40.0%)				
Choronicity^*^			<0.001			
Dichorionic	185 (54.3%)	2,249 (76.3%)			Ref.	
Monochorionic	156 (45.7%)	698 (23.7%)		2.566	1.991–3.307	<0.001
Height (cm)^*^			0.013			
<160	112 (36.1%)	860 (30.3%)				
160–169	194 (62.6%)	1,869 (65.9%)				
≥170	4 (1.3%)	109 (3.8%)				
Pre BMI (kg/m^2^)			0.735			
<18.5	51 (17.6%)	438 (16.3%)				
18.5–23.9	183 (63.1%)	1,787 (66.4%)				
24.0–27.9	44 (15.2%)	370 (13.7%)				
≥28.0	12 (4.1%)	97 (3.6%)				
Weight Gain (kg)^*^			<0.001			<0.001
<10	55 (18.5%)	134 (4.9%)		4.319	2.931–6.364	<0.001
10–14.9	78 (26.3%)	518 (19.1%)		1.476	1.073–2.030	0.017
15–19	104 (35.0%)	1,041 (38.4%)			Ref.	
≥20	60 (20.2%)	1,017 (37.5%)		0.558	0.400–0.779	0.001
HDP^*^			0.034			
No	305 (89.4%)	2,567 (87.1%)				
GH	4 (1.2%)	5 (0.2%)				
HTN	2 (0.6%)	101 (3.4%)				
PE	30 (8.8%)	274 (9.3%)				
ICP^*^			0.097			
Yes	14 (4.1%)	192 (6.5%)		0.533	0.295–0.964	0.037
No	327 (95.9%)	2,755 (93.5%)			Ref.	

* *Factors assigned to multivariate logistic regression analysis*.

*Ref., reference; PTB, preterm birth; ART, assistant reproductive technology; BMI, body mass index; HDP, hypertensive disorders of pregnancy; HTN, hypertension; GH, gestational hypertension; PE, preeclampsia; ICP, intrahepatic cholestasis of pregnancy*.

There was no significant difference in pre-pregnancy BMI and proportions of different pre-pregnancy BMI categories between patients with or without preterm birth before 32, 34, and 37 weeks of gestation. However, some factors associated with reduced risk of preterm birth were also identified by logistic regression analysis. Maternal height ≥170 cm appeared to be a protective factor for preterm birth before 37 weeks (OR 0.642, 95% CI 0.424–0.973, *P* = 0.037). Moreover, the risk for preterm birth before 32 weeks in patients with GWG between 10 and 15 kg were not significantly different compared with those with GWG between 15 and 20 kg. In contrast, GWG above 20 kg was associated with a decreased risk for preterm birth before 32 (OR 0.558, 95% CI 0.400–0.779, *P* = 0.001) and 34 weeks (OR 0.667, 95% CI 0.524–0.849, *P* = 0.001). Intrahepatic cholestasis of pregnancy (OR 0.533, 95% CI 0.295–0.964, *P* = 0.037) was associated with a decreased risk of preterm birth before 32 weeks. The incidence of AFLP and autoimmune diseases were too low to be interpreted by logistic regression due to low event rates.

## Discussion

Our study reports on one of the largest cohorts involving short-term perinatal outcomes with the evaluation of risk factors for preterm birth in twin pregnancies in a Chinese population. The rate of twin pregnancy was 3.3% and preterm birth before 37, 34, and 32 weeks among this population were 62.1, 18.8, and 10.4% respectively. No maternal demise occurred, and the perinatal death rate within 7 days after delivery was 0.7%. The short-term maternal and fetal outcomes were described. Monochorionicity and GWG <10 kg were two major risk factors of preterm birth before 32, 34, and 37 weeks. PE and ICP were associated with increased risk of preterm birth before 37 weeks, whereas maternal age younger than 25 years was a risk factor of preterm birth before 32 and 34 weeks of gestation. Moreover, monochorionicity was associated with a 3-fold increase in the risk of spontaneous and iatrogenic preterm birth before 32 and 34 weeks of gestation.

The preterm birth rate in twin pregnancy has increased significantly in the last three decades as the prevalence of twin births has increased around the world (1, 3, 11, 29). A previous study reported that the twin rate of 2.17% in 2014 in the Chinese population, increased by 32.3% in seven years (3). In addition, the overall preterm birth rates at various cut-off gestational ages, mean neonatal birth weight, and average gestational age were comparable with previous data (11, 30). The national data from the United States revealed that the mean gestational age was 35.3 weeks with a mean birth weight of 2,336 g in twins, while the percentages of preterm birth <37 and 32 weeks of gestation were 58.8 and 11.4%, respectively (11). Similarly, a study from China reported an average gestational age of 35.8 weeks and a mean birth weight of 2,384 g. The preterm birth rate before 37 weeks was 55.5%. Moreover, the methods of delivery (cesarean section: 90.5% and vaginal delivery: 9.4%), and percentage of low birth weight (<2,500 g, 52.7%) (30) were also comparable with our results.

The prevalence of maternal complications including HDP, GDM, ICP, AFLP, hypothyroidism, and postpartum hemorrhage among twin gestations in a Chinese population has seldom been presented. The large cohort size of the current study provides robust reference data on maternal complications that could be applicable in clinical practice. Our study revealed a relatively high rate of these complications, which was consistent with earlier studies concerning pregnancy outcomes in multiple gestations (31, 32). In a larger cohort study published previously, the incidence of PE and postpartum hemorrhage were 9.87 and 8.74%, respectively (31). By contrast, the incidence of PE and hemorrhage in our study were 8.7 and 3.3%, respectively. Meanwhile, 15.7% of our patients were diagnosed with GDM, which was also similar to another study (33). Another study from China focused on perinatal outcomes among twin pregnancies complicated with ICP reported a 6.7% of prevalence, which is closed to the prevalence of 6.3% in our study (34). The incidence of AFLP in twin pregnancies was unclear, whereas the reported incidence in overall pregnant women was ~1:7,000 to 1:15,000 (35), which was much lower than our result (1/822, 0.12%). However, a study from Southwest Wales reported a similarly high incidence of AFLP of approximately 1: 1,000 (36). As these complications can also contribute to adverse pregnancy outcomes (7–9, 25, 37, 38), closer follow-up and monitoring for women with twin pregnancies should be highlighted.

In our study, monochorionicity was significantly associated with preterm birth in twin pregnancy, which approximately doubled the risk for preterm birth in all gestations. The result was in accordance with a systematic review including 29,864 twin pregnancies published in 2020 (14). In this review, monochorionicity significantly increased the risk of preterm birth at ≤ 28 (OR 2.14, 95% CI 1.52–3.02), ≤ 32 (OR 1.55, 95% CI 1.27–1.89), ≤ 34 (OR 1.47, 95% CI 1.27–1.69), and <37 (OR 1.66, 95% CI 1.43–1.93) weeks. Several previous studies have shown that monochorionicity is associated with a higher risk of preterm birth, stillbirth, and perinatal mortality compared with dichorionic twins (39–42). The NICE guidelines recommend planned delivery for uncomplicated monochorionic diamniotic twin pregnancy from 36 weeks (43) and others suggest as early as 34 weeks and certainly <37 weeks (44, 45). Recommendations for early delivery for monochorionic twins may contribute to the increased preterm birth rate due to iatrogenic prematurity. However, a study also revealed that monochorionicity itself has a high association with spontaneous preterm birth (14). In our study, subgroup analysis was conducted to evaluate the effect of chorionicity on spontaneous and iatrogenic preterm birth before 32 and 34 weeks of gestation. Our results demonstrated that monochorionicity was not only associated with a 3-fold increase in the risk of iatrogenic preterm birth but also in the risk of spontaneous preterm birth, suggesting that monochorionicity might be a potential independent predictor of preterm birth in twin pregnancy. Since monochorionicity is also associated with increased fetal risks (46), emergency plans for accidental preterm births should be prepared in the early third trimester in addition to planned deliveries.

Studies evaluating the relationship between pre-pregnancy BMI and pregnancy outcomes produced inconsistent results in twin pregnancies (47–51). Being underweight was considered as a risk factor for preterm birth before 32 weeks, rather than being overweight or obese (47, 48). Maternal complications, such as GDM, PE, and postpartum hemorrhage, were reported to be associated with high pre-pregnancy BMI (49, 52). In our study, pre-pregnancy BMI was not associated with preterm birth at 32, 34, and 37 weeks of gestation. A previous study in Chinese twin pregnancies also revealed no significant difference between normal weight and underweight women regarding spontaneous preterm birth before 37, 35, and 32 weeks (53). This may be partly explained by the fact that the pre-pregnancy BMI in 66.1% of the patients was normal and only 3.7% could be defined as obese according to Chinese categories. Nonetheless, only 6.3% of our patients had a GWG of <10 kg, the lower limit that is adequate for patients in different pre-pregnancy BMI categories according to recommendations from the Institute of Medicine (IOM) and Chinese recommendations for GWG during pregnancy (54, 55). These findings highlighted the importance of maintaining normal weight before conception for better pregnancy outcomes.

GWG has been considered as a predictor in pregnancy outcomes in both singletons and multiple gestations in previous studies (15, 16, 56, 57). Women with GWG below the recommendations of the IOM were at higher risk of small for gestation age and preterm birth, while GWG above the recommendations was associated with decreased risk for preterm birth (15, 16, 56). However, the impact of GWG and pregnancy outcomes were mainly based on patients from western countries and the results were inconsistent in twin pregnancies (50). The IOM lacks recommendations for underweight women (54). In addition, sample sizes of Chinese studies were relatively small and the impact of GWG on preterm birth was inconsistent (16, 58). Lin et al. found that the risk for patients with GWG below the IOM recommendations was 3.55 times risk for preterm birth before 37 weeks and 2.63 times risk for preterm birth before 35 weeks in Chinese twin pregnancies (16), but this study excluded underweight women. Research from Wang et al. only included dichorionic women within normal weight and revealed GWG in accordance with IOM recommendations may reduce the risk of PTB before 32 weeks (58). Nonetheless, the sample sizes of these studies did not exceed 1,000 women, and chorionicity and other potential risk factors for preterm birth were not considered. The recommendations of GWG during pregnancy for Chinese women still lack consensus and GWG in IOM recommendations for normal, overweight, and obese women are overlapping. Our study did not evaluate whether the GWG was in accordance with the recommendations of the IOM. We separated total GWG into several categories and revealed that a lower GWG was significantly related to preterm birth in twin pregnancies and it had a more pronounced effect in predicting earlier preterm birth. In our study, GWG <10 kg tended to associated with preterm birth before 37, 34, and 32 weeks. In particular, twin pregnancy with GWG <10 kg showed a 2-fold risk for preterm birth before 37 weeks and a 3- and 4-fold risk for preterm birth before 34 and 32 weeks, respectively. The GWG from the IOM and Chinese recommendations for all the categories were also not below 10 kg (54, 55), which is partly comparable to our results. Although patients with preterm birth had less GWG compared to full-term patients could also be partly due to the earlier termination of pregnancy. It may be concluded that inadequate GWG was positively related to preterm birth in accordance with previous studies (15, 16, 56, 57). Meanwhile, GWG above 20 kg was associated with decreased risk for preterm birth before 32 and 34 weeks, but published reports demonstrated that excessive GWG may be related to adverse pregnancy outcomes (56, 57). We proposed a possible cutoff value for GWG when evaluating its effect on preterm birth and reinforced the significance of more effective management of GWG during pregnancy.

The effect of PE, ICP, maternal age, and height on preterm birth among twin pregnancies have been discussed in published reports (13, 17, 34). These risk factors have also been identified in this study. Our analysis also revealed that age <25 years was a risk factor for preterm birth before 34 and 32 weeks, which was consistent with earlier studies (59). Another study revealed that age <18 years seemed to be associated with preterm birth before 37 weeks (17). This indicated the importance of conception at an appropriate age. Advanced maternal age (>35 years) was not associated with a significantly increased risk for preterm birth, which could be explained by regular follow-up during pregnancy. Maternal height <160 cm was a risk factor for preterm birth before 37 weeks in studies from the USA (13, 60). Our study did not identify this risk factor, probably due to the difference in the race. However, maternal height >170 cm was negatively associated with preterm birth before 34 and 37 weeks, which was consistent with previous studies (13, 60). It may indicate that in women of short stature, singleton pregnancy is preferential, particularly in those who plan to receive ART. Preeclampsia, rather than chronic hypertension and gestational hypertension, showed an increased risk for preterm birth before 37 weeks, reminds us that better management of the HDP, and alleviating the disease severity will be useful to reduce the risk of preterm birth. Published research has shown that ICP in twin pregnancies was associated with an increased risk of preterm birth before 37 weeks (OR 4.17, 95% CI 2.47–7.04) and before 35 weeks (OR 1.89, 95% CI 1.26–2.95) (34), which was confirmed by our study. These aforementioned risk factors highlight the importance of evaluating maternal clinical characteristics and the prevention as well as management of pregnancy complications.

Analysis of the potential risk factors for preterm birth before 32 gestational weeks, revealed that ICP appeared to be an independent protective factor, whereas PE had no significant effect on preterm birth. This may be attributed to the etiology and the time of onset of the diseases during pregnancy (38, 61, 62). The occurrence of ICP and PE before 32 weeks was rare (61). Patients with ICP would naturally undergo more rigorous monitoring during pregnancy, thereby improving pregnancy outcomes in this population (63, 64). Previous preterm birth history has been considered one of the strongest risk factors for subsequent preterm birth (12, 17). However, preterm birth history did not show a statistically significant association with subsequent twin preterm birth in our study. The probable reasons may be the uncertain gestational weeks, singleton, or multiple pregnancies, and whether it was spontaneous or iatrogenic preterm birth in the previous preterm birth. Published studies revealed that preterm birth at an earlier gestational age (65), spontaneous preterm birth (66), and previous multiple gestation preterm birth (12) were associated with a higher risk for subsequent preterm birth in twin pregnancies.

To the best of our knowledge, this is the largest cohort study on risk factors for preterm birth in twin pregnancies in a Chinese population. We demonstrated the similarities and differences of perinatal outcomes and risk factors for preterm birth compared to previous studies. Moreover, unlike some previous studies from China which focused on one factor such as GWG, we comprehensively evaluated potential risk factors and did not restrict our variables with maternal demographic characteristics, one specific complication, or chorionicity. Based on the current findings, modified counseling, and follow-up strategies for preterm birth can be implemented very early in pregnancy to optimize perinatal outcomes.

The main strength of this study is the large cohort size involving multi-centers in different districts in China. We revealed a positive association between chorionicity, inadequate GWG, PE, ICP, maternal age, height, and preterm birth in twin pregnancies. However, the study also has some limitations. First, the retrospective nature and missing data in some patients may affect the validity of the analysis. Second, this study lacks long-term follow-up of maternal and neonatal outcomes. Further studies, especially prospective trials with long-term follow-up in this field, are warranted.

## Conclusion

We presented the clinical characteristics, complications, and short-term maternal-fetal outcomes in Chinese twin pregnancies. The perinatal outcomes among this population are satisfactory despite a relatively high preterm rate. Monochorionicity and GWG <10 kg were two risk factors for preterm birth before 32, 34, and 37 weeks, whereas maternal age, PE, and ICP were also risk factors for preterm birth at specific gestational ages. Monochorionicity was associated with an increased risk of both spontaneous and iatrogenic preterm birth before 32 and 34 weeks of gestation.

## Data Availability Statement

The original contributions presented in the study are included in the article/Supplementary Material, further inquiries can be directed to the corresponding author/s.

## Ethics Statement

This retrospective birth registry study was approved by the Ethics Committee of Peking Union Medical College Hospital (approval number: PUMCH-JS-1151). The need for informed consent was waived due to the retrospective nature of the study.

## Author Contributions

SL wrote the manuscript and participated in data analysis. JHe, YT, XL, and YC conducted data collection and quality control at their medical centers. JHu and XC participated in data analysis. JG conceived and designed the study. JL participated in designing the study and revising the language. All authors contributed to the article and approved the submitted version.

## Conflict of Interest

The authors declare that the research was conducted in the absence of any commercial or financial relationships that could be construed as a potential conflict of interest.
