# Supplementary material for: Perinatal Outcomes and Risk Factors for Preterm Birth in Twin Pregnancies in a Chinese Population: A Multi-center Retrospective Study
Source: Front Med (Lausanne). 2021 Apr 21;8:657862. doi: 10.3389/fmed.2021.657862 (PMC8096908; doi:10.3389/fmed.2021.657862)
Supplement: Supplementary file 1 [file Table_1.DOCX]

|  | N (Percentile) |  | N (Percentile) |
| --- | --- | --- | --- |
| Age (y) |  | AFLP | 4 (0.1%) |
| < 25 | 186 (5.7%) | AID | 9 (0.3%) |
| 25-34 | 2428 (73.8%) | SLE | 5 |
| ≥ 35 | 674 (20.5%) | SS | 1 |
| Height (cm) |  | Mixed CTD | 1 |
| < 160 | 972 (30.9%) | RA | 1 |
| 160-169 | 2063 (65.5%) | Unspecified | 1 |
| ≥ 170 | 113 (3.6%) | PROM | 404 (12.3%) |
| Prepregnancy BMI (kg/m^2^) |  | Term PROM | 62 |
| < 18.5 | 489 (16.4%) | Preterm PROM | 342 |
| 18.5-23.9 | 1970 (66.1%) | Placenta previa | 96 (2.9%) |
| 24.0-27.9 | 414 (13.9%) | Lower | 20 |
| ≥ 28 | 109 (3.7%) | Marginal | 19 |
| Weight Gain (kg) |  | Partial | 20 |
| <10 | 189 (6.3%) | Complete | 34 |
| 10-14.9 | 596 (19.8%) | Unspecified | 3 |
| 15-19.9 | 1145 (38.1%) | Placenta abruption | 27 (0.8%) |
| ≥ 20 | 1077 (35.8%) | Viral hepatitis | 89 (2.7%) |
| Umbilical cord abnormality | 399 (12.1%) | Hepatitis B | 86 |
| cord around neck | 391 | Hepatitis C | 3 |
| prolapse of cord | 8 | Arrhythmia | 6 (0.2%) |

Table S1: Other maternal clinical characteristics and outcomes

Abbreviations: BMI, body mass index; AFLP, acute fatty liver of pregnancy; AID, autoimmune diseases; SLE, systemic lupus erythematosus; SS, Sjogren syndrome; mixed CTD, mixed connective tissue diseases; RA, rheumatic arthritis; PROM, premature rupture of membrane.
