# Supplementary material for: Perinatal Outcomes and Risk Factors for Preterm Birth in Twin Pregnancies in a Chinese Population: A Multi-center Retrospective Study
Source: Front Med (Lausanne). 2021 Apr 21;8:657862. doi: 10.3389/fmed.2021.657862 (PMC8096908; doi:10.3389/fmed.2021.657862)
Supplement: Supplementary file 2 [file Table_2.DOCX]

|  | Spontaneous preterm birth | | | Iatrogenic preterm birth | | |
| --- | --- | --- | --- | --- | --- | --- |
|  | Adjusted OR | 95% CI | *P* value | Adjusted OR | 95% CI | *P* value |
| Chorionicity |  |  |  |  |  |  |
| Dichorionic |  | Ref. |  |  | Ref. |  |
| Monochorionic | 2.390 | 1.636-3.491 | <0.001 | 3.037 | 2.427-3.800 | <0.001 |

Table S2. Adjusted OR of chorionicity in spontaneous and iatrogenic preterm birth before 34 weeks in Chinese twin pregnancies (N = 3288).

Table S3. Adjusted OR of chorionicity in spontaneous and iatrogenic preterm birth before 32 weeks in Chinese twin pregnancies (N = 3288).

|  | Spontaneous preterm birth | | | Iatrogenic preterm birth | | |
| --- | --- | --- | --- | --- | --- | --- |
|  | Adjusted OR | 95% CI | *P* value | Adjusted OR | 95% CI | *P* value |
| Chorionicity |  |  |  |  |  |  |
| Dichorionic |  | Ref. |  |  | Ref. |  |
| Monochorionic | 2.932 | 1.754-4.900 | <0.001 | 2.510 | 1.872-3.367 | <0.001 |

Abbreviations: OR, odds ratio; CI, confidence interval; ART, assisted reproductive technology; BMI, body mass index; GWG, gestational weight gain; HDP, hypertensive disorders of pregnancy; HTN, hypertension; GH, gestational hypertension; PE, preeclampsia; ICP, intrahepatic cholestasis of pregnancy.

Factors included in logistic regression models: chorionicity (dichorioncity or monochorioncity), maternal age (<25, 25-34, ≥35y), maternal height (<160cm, 160-169.9cm, ≥170cm), parity (nulli or parous), preterm birth history (yes or no), methods of conception (natural or ART), pre-pregnancy BMI (kg/m^2^, <18.5, 18.5-23.9, 24-27.9, ≥28.0), GWG (kg, <10, 10-14.9, 15-19.9, ≥20), HDP (No, HTN, GH, PE), ICP (yes or no).
